# Successful treatment with lenalidomide plus chidamide combination therapy in 3 heavily treated patients with non-Hodgkin lymphoma: Three cases report

**DOI:** 10.1097/MD.0000000000022788

**Published:** 2020-10-23

**Authors:** Shaoxuan Hu, Dongmei Zou, Daobin Zhou, Yan Zhang, Wei Wang, Wei Zhang

**Affiliations:** Department of Hematology, Peking Union Medical College Hospital, Chinese Academy of Medical Sciences & Peking Union Medical College, No.1 Shuaifuyuan Wangfujing, Dongcheng District, Beijing, China.

**Keywords:** chidamide, lenalidomide, non-Hodgkin lymphoma, refractory, relapse, targeted agents

## Abstract

**Rationale:**

The prognosis of patients with aggressive relapsed or refractory (R/R) non-Hodgkin lymphoma (NHL) remains poor. Both immunomodulatory drugs and histone deacetylase inhibitors have demonstrated activity against R/R NHL; yet, the combination of these 2 targeted therapies has rarely been explored.

**Patient concerns:**

Here, we report 3 cases. Case 1 was a 68-year-old woman who presented to our hospital with dyspnea. Case 2 was a 75-year-old man with massive upper gastrointestinal bleeding. Case 3 was a 62-year-old woman with cough, dyspnea, and lymphadenopathy.

**Diagnosis:**

The biopsy results revealed diffuse large B cell lymphoma (DLBCL), DLBCL, and angioimmunoblastic T-cell lymphoma, for Case 1, 2, and 3 respectively.

**Intervention:**

All 3 patients experienced relapse after first-line therapy and multiple lines of salvage therapy. Finally, they all received lenalidomide combined with chidamide.

**Outcomes:**

All 3 patients achieved complete and durable remission. Case 1 relapsed again after 3 months, while the other 2 cases remained in complete remission.

**Lessons:**

To our knowledge, this is the first report of lenalidomide combined with chidamide for the treatment of R/R NHL. Our findings warrant further evaluation of this novel chemo-free therapy in future prospective clinical trials.

## 1 Introduction

Treatment outcomes for patients with aggressive relapsed or refractory (R/R) non-Hodgkin lymphoma (NHL) remain unsatisfactory. Chemotherapy remains the mainstay of treatment, with consolidative autologous stem-cell transplant (auto-SCT) for patients who respond to chemotherapy. Patients who are refractory to salvage chemotherapy or ineligible for intensive therapy and stem-cell transplantation have a poor prognosis, with an expected survival duration of <1 year.^[1]^ Over the recent decade, a number of novel targeted agents have developed for the treatment of R/R NHL, including monoclonal antibodies, tyrosine kinase inhibitors, histone deacetylase inhibitors (HDACi), and immunomodulatory drugs (IMiDs). These agents have demonstrated activity against a variety of subtypes of NHL either as single agents or in combination therapy.^[2]^

Lenalidomide is an IMiD that has demonstrated clinical activity in multiple types of indolent and aggressive NHL. Clinical trials have reported objective response rates (ORRs) of 22% to 35% with single-agent lenalidomide among patients with aggressive R/R B-cell or T-cell NHL.^[3–5]^ Its mechanisms of therapeutic action include activation of the immune system, anti-angiogenesis and direct anti-proliferative, and antineoplastic effects.^[6]^

Chidamide, an oral selective HDACi, is the first new drug approved for the treatment of R/R peripheral T-cell lymphoma (PTCL) in China. Chidamide as single-agent therapy resulted in an overall response rate (ORR) of 28% among patients with R/R PTCL.^[7]^ In preclinical studies, it also exhibited therapeutic effect against B-cell lymphoma cell lines, including anti-proliferative and pro-apoptotic effects.^[8]^ Similar to lenalidomide, it exerts its antitumor action through multifaceted mechanisms such as the induction of cell cycle arrest, apoptosis, and augmentation of anti-tumor immune responses.^[9]^

The combination of an IMiD and HDACi for the treatment of NHL has not been studied extensively. Recently, a preclinical study has revealed synergistic anti-tumor effects between lenalidomide and an HDACi (romidepsin) in lymphoma cell lines.^[10]^ Yet, the clinical efficacy and safety of the combination of these 2 classes of drugs remain largely unknown. Herein, we report 3 cases of patients with aggressive R/R NHL who achieved complete and durable remission when treated with lenalidomide plus chidamide combination therapy.

## 2 Case presentation

### 2.1 Case 1

**Figure 1:**
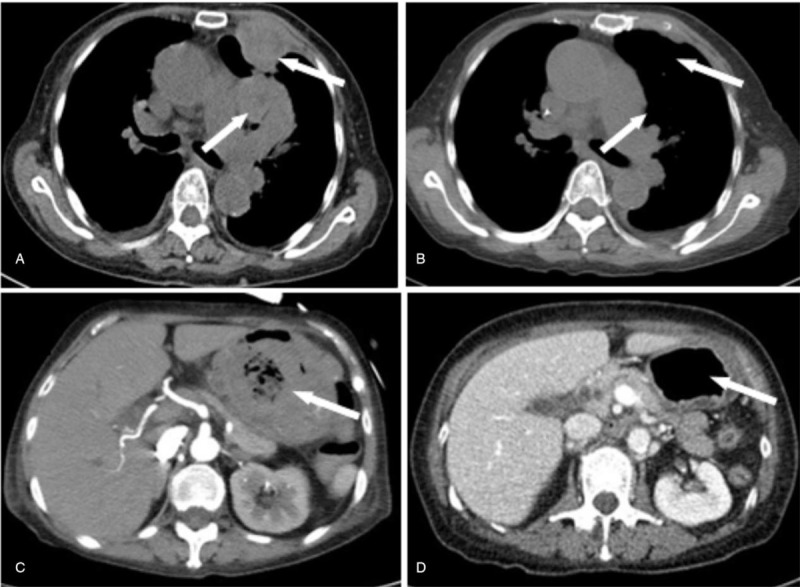
Chest and abdominal CT scan showing multiple pulmonary, pleural, and gastric lesions. A and B, in Case 1 upon the last progression, the largest one with a diameter of 7.7 cm, which disappeared. C and D, After lenalidomide plus chidamide therapy. CT = computed tomography.

This patient was a 68-year-old woman who complained of dyspnea for 2 months and was diagnosed with diffuse large B-cell lymphoma (DLBCL) in February 2012. A positron emission tomography (PET) scan performed at diagnosis revealed multiple neoplastic lesions in the mediastinum and pleura. Immunohistochemical analysis of the pleural biopsy tissue yielded the following findings among lymphoma cells: CD20(+++), CD10(–), Bcl-2(–), Bcl-6(–), Mum-1(–), CD5(+), P53(–), and Ki-67: 70%. A fluorescence in situ hybridization (FISH) test yielded negative results for *MYC* and *BCL-2* rearrangement. A bone marrow biopsy and cerebrospinal fluid cytology test at diagnosis yielded negative results for a malignancy. The patient had a history of non-obstructive hypertrophic cardiomyopathy, with a left ventricular ejection fraction of 66%. She was initially treated with 2 cycles of R-CHOP (rituximab, cyclophosphamide, epirubicin, vincristine, prednisone) followed by 6 cycles of R-CHEP (rituximab, cyclophosphamide, etoposide, vincristine, prednisone) (epidoxorubicin was switched to etoposide from the 3rd cycle due to worsening cardiac function) and achieved complete remission. However, 2 months after the completion of first-line therapy, the patient developed multiple subcutaneous nodules on her chest and abdominal wall, and relapse of DLBCL was confirmed via subcutaneous nodule biopsy. She then received multiple lines of salvage therapy, including R2-GDP (rituximab, lenalidomide, gemcitabine, cisplatin, and dexamethasone), ibrutinib monotherapy and she participated in a clinical trial where she received a spleen tyrosine kinase inhibitor (HMPL-523). Despite the salvage therapies, the disease relapse continued to occur. Upon the last progression, this patient had lymphoma involvement of the gastric wall in addition to the lung, pleura, and mediastinum (Fig. 1), and suffered from diffuse gastric bleeding despite conservative therapy.

After multiple failures of prior therapies, she was started on lenalidomide (25 mg orally every other day) combined with chidamide (20 mg orally twice a week) on the 5th of January 2017. She showed a significant improvement in gastric bleeding and dyspnea as well as marked shrinkage of the subcutaneous nodules after 1 month of therapy. Computed tomography (CT) scans of the chest and abdomen after 2 months of treatment revealed dramatic shrinkage of the pulmonary and pleural nodules and significant improvement in gastric wall thickening (Fig. 1). Treatment was well tolerated, and no side effects were noted. A PET–CT scan performed in October 2017 confirmed a complete response (CR). The patient remained in CR for another 3 months on continuous lenalidomide plus chidamide therapy before subcutaneous relapse recurred in January 2018. The patient then received obinutuzumab (a second-generation anti-CD20 monoclonal antibody) with no response achieved. At the time of this writing, the patient completed chimeric antigen receptor T-cell therapy and achieved a partial response.

### 2.2 Case 2

**Figure 2:**
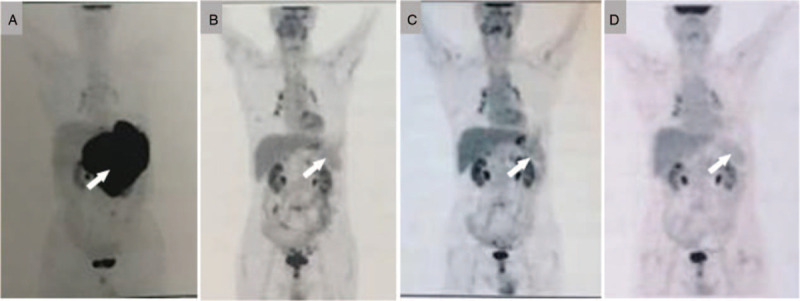
PET/CT scans of Case 2 during treatment: A, prior to initial treatment; B, partial response after 4 cycles of first-line chemotherapy; C, disease progression after 3 months of lenalidomide maintenance therapy; D, CR after 5 months of lenalidomide plus chidamide therapy. CT = computed tomography; CR = complete response; PET = positron emission tomograph.

This patient was a 75-year-old man who presented to our hospital with massive upper gastrointestinal bleeding in December 2016. Endoscopy revealed a giant gastric ulcer, and pathological examination of the gastric biopsy specimen confirmed the diagnosis of DLBCL. Immunohistochemical analysis yielded the following results for neoplastic cells: CD20(+), CD10(–), Bcl-2(90%+), Bcl-6(60%+), Mum-1(60%+), C-MYC(30%+), CD5(scattered cells+), and Ki-67: 90%. A FISH test yielded negative results for *MYC*, *BCL-2*, and *BCL-6* rearrangement. An initial PET/CT scan revealed diffuse gastric wall thickening and multiple abdominal nodules with high fluorodeoxyglucose uptake , with the maximum standard uptake value (SUV_max_) to be 16.8 (Fig. 2A). A bone marrow smear and biopsy tests at diagnosis yielded negative results for malignancy. The patient's past medical history was unremarkable. He was initially treated with 2 cycles of R-CHO (rituximab, cyclophosphamide, epirubicin, vincristine) followed by 2 cycles of R-CHO plus lenalidomide (25 mg every other day on days 1–14 of each 21-day cycle) with a partial response (Fig. 2B). However, treatment was complicated by repeated life-threatening massive gastric hemorrhages and pneumocystis pneumonia. Given his poor tolerance to chemotherapy, he was subsequently switched to a chemo-free therapy with rituximab (375 mg/m^2^ on day 1) and lenalidomide (25 mg every other day on days 1–14 of each 21-day cycle) for 3 cycles, followed by lenalidomide maintenance therapy. However, CR was never achieved, and after 3 cycles of lenalidomide maintenance therapy, the patient developed disease progression (Fig. 2C). Due to the patient's age and clinical condition, he was then treated with chidamide (20 mg twice a week) in combination with lenalidomide (25 mg every other day) starting from November 2017. Treatment was well tolerated, and the patient reported improvement in his appetite and performance status. Four months after commencement of this combination therapy, a PET/CT scan showed complete remission of the tumor lesions (Fig. 2D). He has been on lenalidomide plus chidamide for 7 months so far, without any symptoms or evidence of disease progression.

### 2.3 Case 3

**Figure 3:**
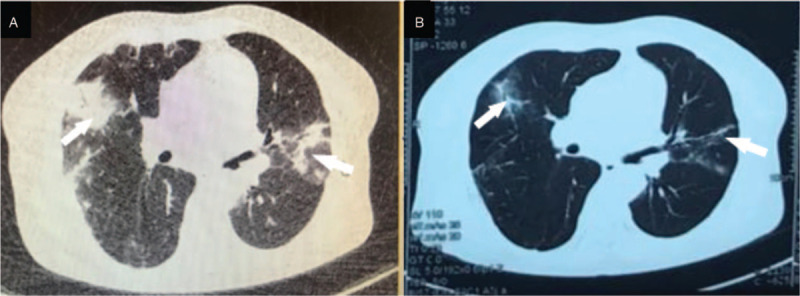
Chest CT scan showing multiple pulmonary lesions in Case 3. A, They all disappeared. B, After lenalidomide plus chidamide therapy. CT = computed tomography.

A 62-year-old woman presented to our hospital with cough, dyspnea, and lymphadenopathy in August 2016. A PET/CT scan revealed bilateral multiple pulmonary nodules with high fluorodeoxyglucose uptake (SUV_max_ 23.2), as well as diffuse lymphadenopathy in the cervical, axillary, mediastinal, abdominal, and inguinal regions. A pathological diagnosis of angioimmunoblastic T-cell lymphoma (AITL) was established based on the biopsy results of the axillary lymph nodes. A bone marrow smear and biopsy revealed no evidence of lymphoma infiltration. The patient's past medical history was unremarkable. After initial treatment with 4 cycles of CHOP (cyclophosphamide, epirubicin, vincristine, prednisone) in combination with chidamide (20 mg orally twice a week), the patient showed partial regression of lymphadenopathy but developed progression of pulmonary lesions. He was then treated with 3 cycles of salvage chemotherapy plus chidamide, and with this he achieved a partial remission and went on to receive high-dose chemotherapy with auto-SCT in March 2017. However, a post-transplant CT scan of the chest still showed multiple residue disease lesions in the lungs bilaterally. After auto-SCT, the patient continued chidamide (20 mg twice a week) as maintenance therapy for 9 months before disease progression in the lung was again observed on a CT scan in December 2017 (Fig. 3A). Upon progression, the patient began to receive lenalidomide 25 mg every other day in addition to chidamide (20 mg twice a week). The patient's symptoms of cough and dyspnea significantly improved upon starting this combination therapy. Five months later, a chest CT scan revealed almost complete remission of the bilateral pulmonary lesions (Fig. 3B). At the time of this writing, the patient remains in good condition with no evidence of disease progression.

Tumor responses were assessed according to the criteria proposed by the International Workshop.^[11]^ The National Cancer Institute Common Terminology Criteria for Adverse Events version 4.0.3 was used for grading adverse events (AEs).

A summary of the clinical characteristics and treatment outcomes of the 3 cases are presented in Table 1.

**Table 1:**
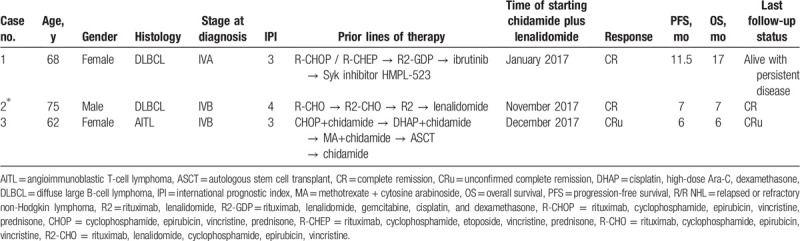
Summary of 3 cases of relapsed/refractory non-Hodgkin lymphoma patients treated with lenalidomide combined with chidamide.

## 3 Discussion

So far, there has been a scarcity of data in the published literature regarding the combination of IMiDs and HDACi for the treatment of R/R NHL. This is the first report of lenalidomide combined with chidamide therapy for patients with aggressive R/R NHL. Our experience demonstrated that this combination therapy achieved excellent outcomes in heavily treated patients with aggressive B-cell and T-cell NHL with a favorable safety profile.

Lenalidomide and HDACi both have antitumor effects on B cell and T cell lymphoma, with synergistic effects on the T cell lymphoma cell line.^[8,10]^ Thus, we treated the patients with lenalidomide and chidamide. The patients were too old and weak to tolerate the regular dose of lenalidomide, and we only had the 25 mg dose form. Therefore, we treated them with 25 mg qod. The dose of chidamide was 20 mg biw according to the medicine specification.

The efficacy of lenalidomide or chidamide as single-agent therapy for R/R NHL was modest in the published literature. In phase 2 clinical trials, the ORR and CR rates of lenalidomide monotherapy were 19% and 28% and 7% and 12% for patients with R/R DLBCL, and 22% and 11% for patients with R/R PTCL, respectively.^[3–5]^ Some investigators reported a higher response to lenalidomide in R/R DLBCL patients with the non-germinal center B-cell-like (non-GCB) phenotype than in those with the germinal center B-cell-like (GCB) phenotype.^[12]^ Similarly, chidamide monotherapy exhibited an ORR of 28% and a CR rate of 14% in a phase 2 study of patients with R/R PTCL, with a median progression-free survival of 2.1 months.^[7]^ In contrast, the combination of lenalidomide with chidamide induced rapid and complete responses in all 3 patients with R/R DLBCL or PTCL even after multiple lines of prior therapy, and the responses were durable. Moreover, all 3 patients failed either lenalidomide or chidamide during their previous treatment. These observations suggest that lenalidomide and chidamide may have a synergistic anti-lymphoma effect that could translate into enhanced clinical efficacy, and therefore this combination therapy might help patients overcome drug resistance to a single-agent therapy with either lenalidomide or chidamide alone.

In support of these assumptions, a recent preclinical study has demonstrated a synergistic effect between lenalidomide and another HDAC inhibitor (romidepsin) in T-cell lymphoma cell lines.^[10]^ In this study, combination treatment with lenalidomide and romidepsin significantly enhanced cytotoxicity in T-cell lymphoma cell lines compared with treatment with either agent alone. Interestingly, sequential treatment with lenalidomide and romidepsin did not show synergistic effects, which further confirms the synergy between these 2 drugs as combination therapy. This enhanced cytotoxic effect was mediated by a variety of anti-proliferative and pro-apoptotic mechanisms including increase in reactive oxygen species production, activation of caspase-8, -9, -3, and poly adenosine diphosphate-ribose polymerase (PARP), induction of endoplasmic reticulum stress, activation of the unfolded protein response sensors, and downregulation of the AKT, MAPK/ERK, and STAT3 pathways.^[10]^

In addition to cytotoxicity, lenalidomide and HDACi possess overlapping yet not identical immunomodulatory effects, which might produce synergy in anti-tumor immunity. For instance, HDACi can augment the immunogenicity of tumor cells by upregulating the expression of major histocompatibility complex class I and II proteins and costimulatory molecules.^[13]^ Consistently, lenalidomide can promote uptake of tumor antigens by dendritic cells and increase the efficiency of antigen presentation to naive CD8+ T cells.^[6]^ In addition, both drugs can enhance the proliferation and cytotoxic effect of T cells against NHL and stimulate cytokine production in the tumor microenvironment including interferon-γ, tumor necrosis factor-α, and interlukin-2.^[6,13]^ It is worth exploring whether the combination of these 2 drugs may synergize to enhance the host immune responses against NHL.

To our knowledge, there has been only one clinical study in the published literature that evaluated the efficacy of combined IMiD and HDACi therapy for R/R NHL. In a phase I/II study reported by Hopfinger et al,^[14]^ the combination therapy comprising lenalidomide, vorinostat, and dexamethasone was assessed in 8 patients with relapsed/refractory PTCL. In contrast to our findings, that study reported only modest efficacy for this combination therapy, with an ORR of 25% and CR in only one patient. However, there are several important differences between that study and our report, which might account for the inconsistent treatment outcomes. Firstly, the maximal tolerable dose of lenalidomide in that study was only 5 mg per day, which is significantly lower than that used for our patients. The reason for the low tolerable dose of lenalidomide in that study is likely because the investigators defined dose-limiting toxicity as the occurrence of any grade 3/4 toxicity during therapy. The combination of lenalidomide at a higher dose (10–25 mg daily) with vorinostat and dexamethasone was associated with 32% of grade 3/4 thrombocytopenia and 44% of grade 3/4 neutropenia in prior studies of patients with R/R multiple myeloma.^[15]^ Thus, the definition of dose-limiting toxicity in the study conducted by Hopfinger et al^[14]^ would probably preclude the possibility of further dose escalation for lenalidomide. Secondly, the study conducted by Hopfinger et al^[14]^ included only patients with PTCL, whereas our reports included both DLBCL (non-GCB phenotype) and PTCL patients. Interestingly, the only 2 patients who responded to therapy in their study had a histological diagnosis of AITL, which is consistent with the histological diagnosis of the patient with T-cell lymphoma in our report. This observation suggests that the efficacy of this combination therapy might differ between different histological forms of NHL. Thirdly, the mechanism of action of vorinostat is not the same as that of chidamide. While vorinostat is a pan-HDACi, chidamide is a selective inhibitor of class I and IIb HDACs.^[7,16]^ It is possible that the subtle differences in mechanisms of action might result in different interactive effects with lenalidomide in combination therapy. Furthermore, although vorinostat has demonstrated efficacy in patients with cutaneous T-cell lymphoma (CTCL),^[16]^ a single-agent study of vorinostat in patients with R/R PTCL has not been conducted. Thus, it is unclear whether vorinostat is as effective for the treatment of PTCL as it is for CTCL.

None of the patients in our report experienced severe adverse events during this combination therapy, indicating that this combination regimen is well tolerated and has a favorable safety profile. It is noteworthy that patient 2 in our report had experienced severe treatment-related complications (e.g., massive gastric hemorrhage and pneumocystis pneumonia) during first-line chemotherapy, which rendered him ineligible for further intensified therapy and stem-cell transplantation. Yet, this patient achieved a complete and sustainable response to lenalidomide plus chidamide without further treatment-related complications. These observations suggest that this combination therapy could be an alternative and promising chemo-free treatment option for patients who are unfit for intensified salvage chemotherapy and auto-SCT.

However, this series has some limitations including the retrospective nature of it and the small number of cases. In addition, these 3 patients had different lymphoma subtypes. It is therefore difficult to draw a strong conclusion. Thus, a well-designed, larger, prospective study is needed in the future.

In conclusion, our case series presented a novel and promising therapy for patients with aggressive R/R NHL. Although the number of cases was small in our report, the dramatic and prolonged efficacy and favorable safety profile of lenalidomide combined with chidamide warrant prospective clinical trials in the future.

## Author contributions

**Conceptualization:** Wei Zhang.

**Project administration:** Yan Zhang.

**Resources:** Wei Wang.

**Writing – original draft:** Shaoxuan Hu, Dongmei Zou.

**Writing – review & editing:** Daobin Zhou, Wei Zhang.
